# Prevalence of Bullying and Its Co-Occurrence with Aggression and Mental Health Problems among Greek Adolescents Attending Urban Schools

**DOI:** 10.34172/jrhs.2022.73

**Published:** 2021-12-28

**Authors:** Zacharias Kalogerakis, Helen Lazaratou, Alexandra Petroutsou, Giota Touloum, Dimitris Dikeos, Marina Economou, Charalampos Papageorgiou

**Affiliations:** ^1^First Department of Psychiatry, National and Kapodistrian University of Athens, Athens, Greece; ^2^Department of Hygiene, Epidemiology and Medical Statistics, National and Kapodistrian University of Athens, Athens, Greece

**Keywords:** Bullying, Adolescence, Prevalence, Mental health

## Abstract

**Background:** Bullying is one widespread violence type that threatens adolescent’s well-being in family, school, and neighborhood. This study aimed to estimate the percentages of the last 12 months bullying behaviors- types among Greek adolescents, and to identify the associations between these behaviors and adolescents’ aggression and mental health- behavioral problems.

**Study design:** A cross-sectional study.

**Methods:** The sample consisted of 1934 adolescents, attending the second grade of 45 randomly selected public and private high schools and senior high schools, of the Greater Athens Metropolitan Area. Bullying involvement was examined by four questions, evaluating the occurrence and type of bullying. The Buss and Perry Questionnaire and Strength and Difficulties Questionnaire were administrated in order to estimate adolescents’ aggression and mental health-behavioral problems, respectively. Information about adolescents’ individual and family characteristics was also collected.

**Results:** Overall, 18.4% of participants reported bullying involvement at school, as a victim (11.0%), a bully (5.0%), or both (2.4%), while verbal bullying was the most common type. Compared to uninvolved participants, victims were significantly more likely to report emotional symptoms and peer problems, bullies were more likely to report physical aggression, and bully-victims physical aggression, hostility, and lower prosocial behavior.

**Conclusions:** Approximately one out of five adolescents were involved in bullying in the past year at school, reporting aggressive behaviors, emotional problems, and/or social difficulties. Further longitudinal research would increase understanding of the mechanisms of bullying involvement and may lead to preventative interventions promoting positive peer interactions in schools.

## Background

 Bullying at school is a universal and significant public health problem.^1^ It is characterized by repeated, intentional and negative actions exhibited by one or more powerful and social prominent peers towards another peer who is unable to defend himself/herself.^2^ Current studies indicate that the estimation rates of bullying range approximately from 9.0% to 54.0%,^1,3-8^ with this wide variation might be attributed to methodological differences between the aforementioned studies.^9^ According to bullying-involved behaviors rates, a cross-national study identified the victims across countries average of 11.0%, the bullies of 10.0%, and the bully-victims one of 6.0%.^5^ With regard to specific bullying and victimization types, verbal (calling names, teasing) and social/relational (rumors spread) are usually described as the two most common,^10^ while it is not rare at all the co-occurrence of more than one types.^11^

 Concerns about prevalence are magnified by concerns about the multiple and long-term negative outcomes of bullying on children and adolescents. In particular, studies focusing on victims of bullying have shown that victimization is associated with physical and psychological issues,^12^ peer rejection,^13^ as well as behavioral, social^14^ and academic maladjustment,^15^ while meta-analytic reviews provide further evidence for between bullying victimization and mental health problems associations.^16^ Furthermore, given the complex nature of bullying and victimization, it is likely that several factors contribute to the presence of these behaviors. For instance, bullying-victimization has been significantly correlated with many factors related to demographics (such as age, gender and ethnicity),^4,17^ family characteristics (such as family environment, parenting behavior, and household socioeconomic status),^18-20^ and social skills.^21^ Importantly, in two meta-analytic reviews examining the predictors of bullying and victimization, it was evidenced that victimization was predicted by internalizing symptoms, conduct problems, as well as social problems. On the other hand, bullies were more likely to exhibit conduct problems, hold negative beliefs about others, and experience social problems.^18,22^ Bully-victims are generally described as having the aspects of both bullies and victims. Thus, bully-victims were found to be presenting the most significant challenges.^18^

 A large body of literature has also focused on better understanding the mechanisms that drive bullying among adolescents. Most of the studies focus on the role of reactive and proactive aggression in bullying, but the results are still inconclusive. Some studies with older children and adolescents have found that bully-victims differentiate from bullies as they tend to act more aggressively, mainly reactively^23^ but also proactively,^24^ and to report higher levels of hostility and anger among young offenders.^25^ Additionally, beyond the reactive and proactive dichotomy, bully-victims are presented to achieve higher scores than bullies for almost all the aggression motives involving rage, revenge, reward and recreation.^26^ Despite these differences, it is apparent that both bully-victims and bullies have more closely resembled motives and experiences than pure victims or controls.^27^ Nevertheless, pure victims score higher than non-victims on (reactive) aggression.^24^ This can be explained by studies that have indicated positive relations between physical or relational victimization and aggression among early adolescents.^28^ Similarly, early victimization has been found to predict later aggressive behavior,^14^ and hostility seems to mediate the relation between prior bullying victimization and subsequent bullying perpetration.^29^

 The present cross-sectional study aimed to estimate the percentage of bullying behaviors (being a victim, bully, or bully-victim) and bullying types (verbal, physical social/relational) among Greek adolescents over the period of the last 12 months. Furthermore, there was a special interest in identifying possible associations between bullying behaviors, mental health- behavioral problems and aggression.

## Methods

###  Procedures and participants 

 The sample consisted of students attending the second grade of 45 (37 state and eight private) High Schools and Senior High Schools of the greater Athens Metropolitan area in Greece. The school selection involved a five-region geographical stratification and a random school sampling within each region- stratum. Ethics approval was granted by the Greek Ministry of Education, Research and Religious Affairs and informed consents were obtained from both parents and students prior to participation in the study. From October 2016 to March 2017, 1976 students during a class period and in the presence of class teacher, completed anonymously a test battery (the study had a student response rate of 70.02%). Responding students were excluded if they were out of the target range of age (18 participants were older than 17 years, 0.9%), or if they didn’t answer the bullying- related questions (*N* = 24, 1.2%), yielding a sample of 1934 participants. Of this final sample, 894 (46.2%) were boys and 1040 (53.8%) girls, aged between 12 and 17 years (*M* = 15.35, *SD* = 1.36).

###  Bullying outcomes

 In order to avoid possible terminology misunderstandings, a bullying definition^2,30^ was provided during the survey (highlighting the phenomenon’s characteristics of power imbalance, intention to harm, and repetitiveness), while participants’ anonymity contributed to the elimination of their resistance in reporting bullying involvement. In this context, participants were asked to answer two close-ended and two open-ended questions. The first close-ended question (answered with “Yes” or “No”) was evaluating if they had been bullied during the past 12 months at school and in what way (open- ended question), while the second close-ended question was asking if they had bullied others during the past 12 months at school and in what way (open-ended question). Participants responses to the two close-ended questions lead to four possible bullying-related behaviors: (*a*) victims (those who had been bullied and had not bullied others); (*b*) bullies (those who had bullied others but had not been bullied); (*c*) bully-victims (those who had both been bullied and bullied others); (*d*) uninvolved (those who had not been bullied and had not bullied others). Participants answers to the two open-ended questions were coded through the following labels: (*a*) verbal (taunting, teasing, calling mean names, making fun of); (*b*) physical (pushing, hitting, kicking); (*c*) relational (excluding socially others, spreading rumors).

###  Aggression

 Aggression feelings and behaviors were measured by the Greek adaptation of Buss– Perry Aggression Questionnaire (AQ).^31,32^ The AQ is a 29-item self-report measure, widely used in adolescent populations. It is scored on a 5-point Likert scale from one (“extremely uncharacteristic of me”) to five (“extremely characteristic of me”) with larger scores indicating higher levels of aggression. The AQ yields scores for four sub-dimensions of aggression: physical aggression (assessed by nine items), verbal aggression (assessed by five items), anger (assessed by seven items) and hostility (assessed by eight items).

###  Mental health and behavioral problems

 Adolescent mental health problems and behaviors were assessed by the Greek adaptation of the self-reported version of the Strengths and Difficulties Questionnaire (SDQ).^33-35^ The SDQ is a brief screening questionnaire that contains 25 items describing behavior, each of which is to be rated on a 3-point scale (“not true’’, “somewhat true’’, “certainly true’’). It consists of five subscales of five items each, covering emotional symptoms, conduct problems, hyperactivity/ inattention, peer relation problems and prosocial behavior.

###  Individual and socio-demographic characteristics

 Various participants’ socio-demographic characteristics were assessed. In order to estimate participants household economic status and identify any difficulties in meeting food needs, the Household Food Insecurity Access Scale (HFIAS)^36^ was used. The HFIAS is a self-completed scale assessing the degree of household food insecurity and is composed of a set of nine double questions (occurrence and frequency of occurrence). Three of these questions were included in the present study in order to measure the extent of anxiety- uncertainty about food (“Did you worry that your household would not have enough food?”), insufficient food quality (“Did you or any household member have to eat a limited variety of foods due to a lack of resources?”), and insufficient food intake (“Was there ever no food to eat of any kind in your household because of lack of resources to get food?”). These three questions have been combined into one binary variable (1 = “Presence of at least one condition of household food insecurity’’, 0 = “Absence of household food insecurity’’) because of their high correlation, facilitating the study’s statistical analyses.

###  Statistical analyses

 Descriptive statistics were calculated for initial data analysis. The prevalence of past 12 months bullying behaviors, as well as the percentages of bullying types, was computed. In order to control for the effects of participants’ gender- age- school class/school area on bullying outcomes, three control groups (one for each bullying behavior) were created. These groups were generated by randomly matching (for gender, age, and school class) one to one, the uninvolved participants with the victims, bullies, and bully-victims (cases) participants. Then, chi-square tests were applied to compare differences in individual, socio-demographic, and family characteristics, between cases and the uninvolved/control ones. Similarly, Wilcoxon signed rank tests or paired *t* tests (based on the results of the Kolmogorov-Smirnov continuous variables’ normality tests) were performed to explore differences in aggression and metal health problems between victims, bullies and bully-victims and their matched controls. Multiple logistic regression models (using each one of the bullying-related behavior variables as the dependent one) were conducted with the intention of further examining which of the adolescents’ characteristics/ problems were associated with the corresponding bullying-related behaviors. All analyses were applied using IBM SPSS version 20.0, at 5% level of significance.

## Results

 The majority of participants was Greeks (88.3%), born in Greece (93.9%) and living with both parents (80.5%). Most of participants’ family structure was intact (80.7%), with no difficulties in meeting food needs in their households (81.7%). The majority of participants was right-handed (87.3%) with no last year school failure (98.0%) and was receiving pocket money allowance by their parents (79.5%). According to their responses, a total of 18.4% of the sample reported involvement in bullying behaviors during the past 12 months at school, as a victim (11.0%), a bully (5.0%), or both (2.4%). Verbal bullying was the most common type within all bullying-involved groups (48.6% within victims, 30.9% within bullies, and 40.4% as victims and 29.8% as bullies within bully-victims) followed by physical and relational one, while the verbal-physical type was the most common mixed type (Table 1).

**Table 1:** Frequencies of bullying types among bullying-involved participants

**Bullying type**	**Victims (n=212)**		**Bullies (n=97)**		**Bully-victims**			
					**As bullies (n=47)**		**As victims (n=47)**	
	**Number**	**Percent**	**Number**	**Percent**	**Number**	**Percent**	**Number**	**Percent**
Verbal	103	48.6	30	30.9	14	29.8	19	40.4
Physical	29	13.7	18	18.6	14	29.8	13	27.7
Relational	16	7.5	7	7.2	3	6.4	4	8.5
Verbal-physical	23	10.8	12	12.4	6	12.8	6	12.8
Verbal-relational	12	5.7	4	4.1	0	0.0	2	4.3
Physical-relational	1	0.5	0	0.0	0	0.0	0	0.0
All types	2	0.9	0	0.0	1	2.1	0	0.0
Not specified	26	12.3	26	26.8	9	19.1	3	6.4

 Furthermore, bivariate analysis showed that participants’ birthplace, family structure, living- with status, handedness and past year school failure did not seem to be associated with the occurrence of any bullying behaviors (Table 2). On the other hand, participants’ nationality was associated with being a victim or bully and food deprivation with being a victim or bully-victim, while being a bully was correlated with receiving pocket money. Physical aggression and anger were associated with all bullying involved behaviors, verbal aggression with bullies and bully-victims, and hostility with victims and bully-victims. Regarding mental health problems, conduct and hyperactivity/inattention difficulties were common in all bullying involved behaviors. Particularly, emotional and peer problems were more frequent among victims and bully-victims while prosocial behavior was evident among bully-victims.

**Table 2:** Association between participants’ characteristics, aggression, and mental health– behavioral problems and bullying- involved behaviors

**Variables**	**Victims**				**Bullies**				**Bully-victims**			
	**n**	**%**	**χ** ^ 2 ^	***P *** **value**	**n**	**%**	**χ** ^ 2 ^	***P *** **value**	**n**	**%**	**χ** ^ 2 ^	***P *** **value**
Greek nationality ^a^												
Cases	185	87.3	4.56	0.033	79	81.4	5.56	0.018	37	78.7	3.02	0.082
Uninvolved	198	93.4			90	92.8			43	91.5		
Birthplace (Greece)^a^												
Cases	197	92.9	2.25	0.133	88	90.7	0.65	0.420	40	85.1	1.79	0.181
Uninvolved	204	96.2			91	93.8			44	93.6		
Right- handed ^a^												
Cases	186	87.7	0.60	0.439	71	73.2	3.74	0.053	40	85.1	0.01	1.000
Uninvolved	191	90.1			82	84.5			40	85.1		
Intact family structure^a^												
Cases	179	84.4	0.16	0.693	68	70.1	2.21	0.137	36	76.6	0.25	0.614
Uninvolved	176	83.0			77	79.4			38	80.9		
Living with both parents^a^												
Cases	174	82.9	0.16	0.693	70	72.9	0.64	0.425	34	72.3	0.95	0.330
Uninvolved	177	84.3			74	77.9			38	80.9		
No- family material deprivation^b^												
Cases	156	73.6	9.86	0.002	82	84.5	0.18	0.673	29	61.7	4.21	0.040
Uninvolved	182	85.8			84	86.6			38	80.9		
Receiving pocket money ^c^												
Cases	168	79.2	0.01	0.980	91	93.8	8.70	0.003	38	80.9	0.55	0.458
Uninvolved	167	79.1			77	79.4			35	74.5		
No- past year school failure ^d^												
Cases	209	98.6	1.01	0.315	95	97.9	0.34	0.560	45	95.8	2.04	0.153
Uninvolved	211	99.5			96	99.0			47	100.0		
**Variables**	**Median**	**IQR**	**z**	***P *** **value**	**Median**	**IQR**	**z**	***P *** **value**	**Median**	**IQR**	**z**	***P *** **value**
Physical aggression												
Cases	21.0	17-26	-2.46	0.014	31.0	22-38	-6.08	0.001	26.0	21-33	-3.47	0.001
Uninvolved	19.0	15-24			20.0	15-25			19.0	14-25		
Verbal aggression												
Cases	14.0	12-17	-1.50	0.132	16.0	14-19	-5.23	0.001	15.54	4.61	2.33	0.020
Uninvolved	13.0	11-16			13.0	11-15			13.46	3.81		
Anger												
Cases	20.0	17-25	-2.64	0.008	24.0	18-29	-4.52	0.001	21.83	6.08	2.36	0.018
Uninvolved	19.0	15-23			19.0	14-23			18.71	6.39		
Hostility												
Cases	23.67^e^	6.83^f^	5.99^g^	0.001	21.0	17-27	-1.70	0.089	24.25	7.47	5.03^g^	0.001
Uninvolved	19.84^e^	6.67^f^			20.0	14-23			17.60	5.76		
Emotional symptoms												
Cases	4.0	2-6	-6.12	0.001	2.0	1-4	-1.09	0.276	4.0	2-6	-2.98	0.003
Uninvolved	2.0	1-4			2.0	1-5			3.0	1-4		
Conduct problems												
Cases	3.0	2-4	-3.41	0.001	4.0	2-5	-4.37	0.001	4.0	2-6	-3.50	0.001
Uninvolved	2.0	2-3			2.0	2-3			2.0	2-3		
hyperactivity/ inattention												
Cases	4.0	2-5	-3.58	0.001	5.0	3-6	-3.78	< 0.001	4.94	2.37	3.16^g^	0.002
Uninvolved	3.0	2-5			3.0	1-5			3.28	2.23		
Peer problems												
Cases	3.0	1-4	-6.92	0.001	1.0	0-3	-0.40	0.690	2.0	1-4	-1.99	0.046
Uninvolved	1.0	0-2			1.0	0-2			2.0	1-3		
Prosocial behavior												
Cases	8.0	7-9	-0.81	0.417	8.0	6-9	-1.25	0.211	7.0	5-8	-3.06	0.002
Uninvolved	8.0	6-9			8.0	6-9			8.0	6-9		

Abbreviation: IQR, Interquartile range. ^a^ versus “Other”. ^b^ versus “Family material deprivation”. ^c^ versus “No”. ^d^ versus “Past year school failure”. ^e^ M. ^f^ SD. ^g^ t value of paired *t*-test.

 As Table 3 shows, logistic regression analyses evidenced that victims were significantly more likely to report emotional symptoms OR = 1.14 (95% CI: 1.01, 1.27) and peer problems OR = 1.33 (95% CI: 1.16, 1.52) than uninvolved participants. Similarly, bullies were 3.70 (95% CI: 1.17, 11.73) times more likely to report that they receive pocket money and 1.10 (95% CI: 1.05, 1.16) times more likely to report physical aggression than those uninvolved. Furthermore, bully-victims were more likely to report physical aggression OR = 1.12 (95% CI: 1.01, 1.23) and hostility OR = 1.24 (95% CI: 1.08, 1.42) than uninvolved participants, and less likely than uninvolved to report prosocial behavior OR = 0.73 (95% CI: 0.53, 0.99).

**Table 3:** (Multiple) Logistic regression analyses of participants’ aggression and mental health– behavioral problems as predictors of adolescents’ bulling- involved behaviors

	**Model 1 ** **(Victims vs Uninvolved)**		**Model 2 ** **(Bullies vs Uninvolved)**		**Model 3** **(Bully-victims vs Uninvolved)**	
	**OR (95% CI) **	***P *** **value**	**OR (95% CI)**	***P *** **value**	**OR (95% CI)**	***P *** **value**
Nationality	0.53 (0.25, 1.13)	0.101	0.45 (0.13, 1.49)	0.190	Not included	
Family material deprivation	1.48 (0.85, 2.57)	0.165	Not included		1.44 (0.41, 5.03)	0.568
Pocket money	Not included		3.70 (1.17, 11.73)	0.027	Not included	
Physical aggression	1.01 (0.98, 1.05)	0.465	1.10 (1.05, 1.16)	0.001	1.12 (1.01, 1.23)	0.029
Verbal aggression	Not included		1.07 (0.95, 1.20)	0.280	0.88 (0.72, 1.08)	0.214
Anger	0.96 (0.92, 1.01)	0.146	1.03 (0.95, 1.11)	0.482	0.91 (0.79, 1.04)	0.173
Hostility	1.03 (0.98, 1.08)	0.190	Not included		1.24 (1.08, 1.42)	0.003
Emotional symptoms	1.14 (1.01, 1.27)	0.028	Not included		1.00 (0.73, 1.37)	0.975
Conduct problems	1.18 (0.99, 1.39)	0.054	1.01 (0.77, 1.33)	0.937	1.18 (0.80, 1.73)	0.402
Hyperactivity/ inattention	0.97 (0.87, 1.09)	0.656	1.07 (0.90, 1.26)	0.463	1.12 (0.84, 1.50)	0.442
Peer problems	1.33 (1.16, 1.52)	0.001	Not included		0.76 (0.53, 1.09)	0.133
Prosocial behavior	Not included		Not included		0.73 (0.53, 0.99)	0.045

## Discussion

 Bullying is a widespread public health problem that impacts a large number of young people at school. Within the prevalence range of a large cross-national survey,^5^ our study showed that 18.4% of participants reported bullying-related experiences at school during the last 12 months. In more detail, 11.0% of adolescents mentioned that they were victims of bullying, 5.0% were perpetrators, and 2.4% were both victims and perpetrators, while verbal bullying was the most common type^9^ within all bullying-involved groups and verbal-physical type was the most common mixed type. This percentage of victims is similar to the percentages found in other international^4,5,8,37^ and Greek^38^ studies, while the percentage of bully-victims is close to those reported in other studies.^4,6,39^ The percentage of bullying perpetration found in this study is closer to the one estimated in an Australian meta-analytic review ^3^ compared to the higher estimations found in many other studies^4,5,37-39^. This difference is probably due to methodological issues and, most likely, to the variances in the time frame (from two to 12 months) and setting (in and out of school environments) in which bullying occurred.

 With regard to the demographic characteristics of participants, none (except of receiving pocket money) was found to be associated with the occurrence of bullying behaviors (the effects of gender, age, and school class/ area were preliminary statistically controlled). With the vast majority of participants being Greek and being born in Greece, nationality was not associated with bullying. This finding highlights the importance of a deeper investigation when examining nationality by assessing youths’ ethnic background along with the school classes’ ethnic composition and schools’ minority density.^17,40^ Other relevant individual factors that may add a greater likelihood of school, social or mental health problems in adolescence, such as non-right handedness^41^ and past year school failure,^7,42^ did not seem to correlate with bullying occurrence. Concerning the family-related factors included in this study (intact family structure, living with both parents, household material competence), none of them were found to be significantly associated with victimization or bullying. This is contrary to previous research showing that other contextual factors related to family characteristics are significant correlates of bullying (although some of these correlations were weak or small to moderate).^18-20^ On the other hand, receiving pocket money was the only individual/ family factor that was significantly associated to bullying behavior. This specific variable has not been tested in previous studies and, thus, it is not clear if it is a valid indicator of socioeconomic status. In Tippett and Wolke’s meta-analysis, it was found that bullies were slightly less likely to come from higher SES backgrounds, which is contrary to the finding of this study if pocket money is considered a measure of SES.^19^ One possible explanation for this inconsistency is that Tippett and Wolke used different indicators when referring to SES. It is also possible that receiving a greater amount of pocket money is not a representation of SES but of less parenting monitoring or supervision. Indeed, research has shown that bullying is associated with less parental monitoring.^43,44^ Future research with well-defined measurements assessing multiple individual, family and environmental factors is also needed to disentangle how each factor contributes to bullying involved behaviors.

 The second aim of the present study was to identify which aggressive patterns and mental health problems usually accompany bullying behaviors. Regarding aggression, none of its different aspects estimated in this study to be associated with victims’ aggressive behavior. This is inconsistent with previous research studies that have indicated positive correlations between victimization and aggression.^24,28,29^ One possible explanation for these differences is that previous studies have incorporated measurements of particular theoretical constructs of aggression, such as proactive and reactive aggression,^24^ whereas in this study four sub-dimensions of aggression (physical aggression, verbal aggression, anger, and hostility) were incorporated for the operationalization of aggression. Another important methodological difference between the current study and previous studies is the age of participants. Specifically, the sample of this study is composed of second graders whereas previous research was done mainly with pre-adolescent children.^24,28,29^ Given that pre-adolescence is an important developmental stage with marked changes and challenges (e.g., autonomy from parents, reliance on peer groups, advanced school obligations), it is possible that middle school children are more likely to discharge their inner pressures via aggressive behaviors compared to elementary children who are not exposed to similar stressors. Thus, the lack of an association between victim’s bullying behavior and aggression might be due to developmental reasons. The question that emerges is whether similar results would be obtained with an older age group. Nevertheless, previous research has shown that victims are less likely to report a tendency to aggressiveness compared to the rest types of bullying behaviors (bully and bully-victim).^8^ Therefore, it is possible that the association between aggression and bullying is less common among pure victims. Regarding participants’ mental health issues, bullying victimization was associated with emotional symptoms and problems with peers. Similar associations were also observed with emotional issues (such as depression and anxiety),^16^ internalizing symptoms and peer relationship problems in previous studies.^13,14,18,22^ Although conduct problems did not reach statistical significance in the current study, they had an expectedly^22^ high occurrence among victims.

 Concerning the second type of bullying behavior, pure bully, it was found to be associated with physical aggression. By definition, bullying involves imbalance in power that can be based on differences concerning physical strength.^2,10,27^ This physical dominance combined with widely mentioned high levels of aggression^24^ and low levels of prosocial behavior^45^ are usually apparent in the attitudes of perpetrators. Our data failed to connect bullying behavior with well-established associated factors observed in other studies, like externalizing behaviors^46^ and peer- social problems.^18,22^ These inconsistencies might be attributed to methodological differences across studies. For instance, in Wang and colleagues’ study the term externalizing behavior referred to substance use and carrying weapons,^46^ while in this study it was used as a generic term describing the subscales of behavioral problems and hyperactivity derived from SDQ.^35^ Furthermore, the lack of an association between peer-social problems and bulling behavior might be attributed to the cross-sectional nature of this study, as longitudinal/prospective studies have evidenced the interconnectedness of these two phenomena.^22^ However, it is important to note that antisocial behaviors are not rarely perceived as positive in peer groups, as bullies tend sometimes to be perceived as “popular” and report having many friends.^47^ In line with these findings, some researchers have identified a subgroup of “socially skilled” bullies, characterized by the ability manipulate others and maintain positive relationships with peers.^24^

 Finally, the bully-victim group was the most problematic among the various bullying types of the present study. In line with previous studies who have identified bully-victims as the most aggressive and hostile,^8,23-25,27^ bully-victims were also found to be both physically aggressive and hostile in this study. While physical aggression could be probably explained (as above) by the “bully” dimension of bully-victims behavior, hostility as a cognitive component of aggression, has recently been found to mediate the relation between prior victimization and subsequent bully perpetration.^29^ In other words, hostility provides a link between bullying victimization and perpetration. This could explain the strong association found between hostile-related behaviors and the bully-victim type. Furthermore, among the behavioral- mental health problems examined in the present study, and inconsistently with prior meta-analytic studies,^18,22^ the bully-victim behavior was not found to be associated with conduct and hyperactivity/ inattention difficulties. This could probably be explained, as mentioned above, by the studies’ divergence in methodological approaches of determining/ evaluating the externalizing participants’ problems. On the other hand, a significant association was found with lower levels of prosocial behavior. In general, bully-victims tend to inaccurately believe that their peers intend to harm them,^27^ feel rejected and hold negative attitudes and beliefs about others.^18^ Thus, bully-victims have less or inappropriate interactions with their peers and therefore have few opportunities to develop their prosocial skills and behavior.

 Several limitations of the present study should be noted. First, the data were cross-sectional and as such they preclude us to draw any firm conclusions as to the causal nature of the associations between victimization-bullying behaviors and the studied factors. Second, all measures relied solely on self-report. While self-report is a common and accepted method of measuring bullying, collecting additional information from parents, and teachers would probably minimize subjectivity. Similarly, using a diagnostic tool instead of a screening questionnaire, as well as a specific measurement of proactive and reactive aggression, would have been a better identification of participants’ mental health and aggression traits. Finally, regarding the analysis of covariates, only the most basic demographic variables were included in this study. Other important variables, such as parents’ supervision and support, family SES, and neighborhood context, were not estimated and, thus, the potential of residual confounding cannot be excluded.

## Conclusion

 Bullying is a multidimensional phenomenon that motivates scientific community worldwide. This study provided data on the prevalence and aggression- mental health correlates of bullying among Greek adolescents. The findings indicate that almost one out of five adolescents were involved in bullying in the past year in school. That involvement was associated with aggressive-related behaviors (for bullies and bully-victims), emotional problems (for victims), and social- peer difficulties (for victims and bully-victims). Further prospective/ longitudinal research is needed in order to increase our understanding of the mechanisms that may lead to bullying involvement (including family-cultural influences, neighborhood-school contextual factors and peer interactions) and shed light to the long-term consequences of it. This knowledge could then be used to develop preventative interventions targeting bullying behaviors, which could focus on increasing awareness (mainly of teachers and parents) about bullying and on changing classroom climate by promoting positive peers interactions.

## Acknowledgements

 The authors’ appreciation goes to the adolescents and their teachers who took part in this study.

## Conflict of interest

 The authors declared no potential conflicts of interest with respect to the research, authorship and/or publication of this article.

## Funding

 The authors received no financial support for the research, authorship and/or publication of this article.

**Highlights**

- Almost one in five adolescents are involved in bullying in the past year at school.
- Verbal bullying is the most common type within all bullying-involved groups.
- Aggressive patterns and mental health problems usually accompany bullying behaviors.
